# Family history identifies sporadic schizoaffective disorder as a subtype for genetic studies

**DOI:** 10.4102/sajpsychiatry.v26i0.1393

**Published:** 2020-04-20

**Authors:** Nicolaas J. van der Merwe, Maria Karayiorgou, René Ehlers, Johannes L. Roos

**Affiliations:** 1 Department of Psychiatry, Faculty of Health Sciences, University of Pretoria, Pretoria, South Africa; 2 Department of Psychiatry, Columbia University, New York, United States; 3 New York State Psychiatric Institute, New York, United States; 4 Department of Statistics, Faculty of Natural and Agricultural Sciences, University of Pretoria, Pretoria, South Africa

**Keywords:** schizophrenia, family history, genetic studies, novo genetic events, familial-sporadic distinction

## Abstract

**Background:**

Schizophrenia is a heterogeneous disorder with strong genetic vulnerability. Family history of schizophrenia has been considered in genetic studies under several models. *De novo* genetic events seem to play a larger role in sporadic cases.

**Aim:**

This study used the familial–sporadic distinction with the aim of identifying a more homogeneous phenotype to delineate the genetic and clinical complexity of schizophrenia.

**Setting:**

The study was conducted at Weskoppies Hospital, Pretoria, South Africa.

**Methods:**

The study included 384 participants with schizophrenia or schizoaffective disorder from the Afrikaner founder population in South Africa who are considered comparable to Caucasian patients from the United States. A comprehensive data capturing sheet was completed.

**Results:**

When schizophrenia and schizoaffective disorder diagnoses were considered jointly, we found no significant differences between the sporadic and the familial groups for age at disease onset, season of birth, comorbid diagnoses, clinical symptomatology, history of suicide or marital status. When the diagnoses were examined separately, however, the sporadic schizoaffective disorder, bipolar type, was found to have a significantly lower age at onset (mean 20.6 vs. 25.3 years).

**Conclusion:**

The sporadic schizoaffective disorder, bipolar type, forms a more homogeneous subgroup for genetic studies.

## Introduction

Schizophrenia is considered etiologically as a heterogeneous group of disorders.^1^ Against this background of considerable heterogeneity, the presence of mental illness in close relatives (familial cases) versus no history of mental illness in close relatives (sporadic cases) has been considered in genetic studies.^2,3,4^ This criterion represents one of the multiple strategies used to define a more homogeneous group of subjects.^5^

It is difficult to ascertain familiality accurately, especially in outbred populations where families are largely mobile and historical data on multiple family members are scarce. However, despite the difficulties, it is a variable of fundamental importance in genetic studies, as already demonstrated. Specifically, significantly increased rates of *de novo* copy number variants have been reported in sporadic, as opposed to familial cases of schizophrenia,^1,4^ and *de novo* single-nucleotide polymorphisms are also more common.^1^ This provides evidence for an association on a genetic level, with at least some mutations enriched in sporadic cases.^1^

Numerous attempts to parse the complicated, heterogeneous clinical presentation of schizophrenia into homogeneous groups that may be suitable for genetic research have taken place without much success. A number of clinical as well as environmental factors have been considered, such as gender, birth and perinatal insults, season of birth, early childhood pre-psychotic deviant behaviour, substance abuse, several comorbid conditions, specific clinical symptoms, age at the onset of primary illness^6^ and suicidality,^7^ amongst others.

Phenotype refinement has been beneficial in elucidating the genetic architecture of other neuropsychiatric disorders, such as Alzheimer’s disease^8^ and Parkinson’s disease.^9^ Gene identification efforts in schizophrenia may also benefit through the definition of more homogeneous phenotypes that allow a meaningful sub-grouping of individuals with the disease.^5^

To our knowledge, a phenotype using a familial versus sporadic sub-grouping has not been characterised. The aim of this study was to use this sub-grouping to determine a more homogeneous phenotype for genetic research via comparison of various clinical, socio-demographic as well as environmental variables in familial versus sporadic schizophrenia.

## Methods

### Study design and participants

This study included participants from the collaborative study on Genetics of Schizophrenia led by Dr Karayiorgou since 1997.^10^ Participants were drawn from the Afrikaner founder population in South Africa and were initially recruited from inpatient, outpatient and follow-up clinics at Weskoppies Hospital in Pretoria. Previous work has shown that the Afrikaner sample is clinically comparable to samples recruited from outbred populations, including the United States.^10^ The Afrikaner sample, however, being derived from a founder population, offers additional advantages for mapping genes caused by reduced genetic heterogeneity and a more uniform environment.^10^ The close-knit nature of Afrikaner families and the presence of long-term care mental health facilities in the country allow for a relatively reliable ascertainment of family history of mental illness and subsequent distinction between sporadic and familial groups.

This study made use of a cross-sectional observational design. We used data from the database that has been assembled for the ongoing genetic research on this population. The database contains detailed information obtained by administering in-person the Diagnostic Interview for Genetic Studies (DIGS) to all subjects, medical intake forms, questionnaires on early development, family history, medication history, as well as information derived from witnesses and medical records when necessary.^10^ From this extensive database, we selected a subsample of 384 participants for this study. Our criteria for this selection were: (1) subjects met full Diagnostic and Statistical Manual of Mental Disorders (DSM) criteria for schizophrenia or schizoaffective disorder (as determined by the DIGS, which in multi-site test–retest analyses have been found to be reliable for the major DSM diagnostic categories^11^), and (2) subjects in whom family history of schizophrenia or schizoaffective disorder had been ascertained and was available. Any subject with a first-degree, second-degree or third-degree relative diagnosed with schizophrenia or schizoaffective disorder was considered as a familial case for the analyses presented here.

### Measurements

All data recorded upon the initial recruitment of subjects, as well as any available follow-up data, were reviewed and captured using a data capturing sheet. We assessed basic socio-demographic variables, such as gender, month of birth (grouped to reflect seasonality, with July to September representing the peak months for winter or spring births in the southern hemisphere, previously associated with schizophrenia risk),^12^ marital status, employment status and the highest level of education.

We also assessed several clinical variables, including age at onset of the primary psychiatric diagnosis (defined as the age when full DSM criteria were first met), suicide ideation and number of suicide attempts, as well as the presence of comorbid diagnoses. Amongst the comorbidities, we considered the presence of intellectual disability (ID) or borderline ID, obsessive compulsive disorder or symptoms, major depressive disorder, alcohol abuse, cannabis abuse or use of more than one illicit substance. An illicit substance was defined as any substance with abuse potential and not legal under South African law. Notable legal substances include tobacco and alcohol, with cannabis defined as an illegal substance under current legislation. Several individual clinical symptoms were also considered. These included delusions, hallucinations (captured in three sub-categories of visual, auditory and other sensory hallucinations), disorganised behaviour, disordered thought and alogia.

Information about history of early childhood, pre-psychotic and deviant behaviour before the age of 10, which has been previously linked to the development of schizophrenia,^13^ was also obtained from the database where available. The following seven early deviant behaviours were captured and analysed: social difficulties, extreme aggression, odd behaviour, extreme fears, chronic sadness, learning disability and impairment in attention.^14^

Finally, birth and perinatal insults were also considered.

### Data analysis

Logit models were fitted to test whether there is a relationship between a binary outcome variable and covariates of interest (differential family history, sex and primary diagnosis). Patients for whom the response to the outcome variable was unknown were excluded from the analysis. The binary outcome variables considered in the different models were comorbid diagnosis, cannabis use, illicit substance abuse, a patient being single, perinatal insults, hallucinations, early deviant behaviour and suicidality. Descriptive data is provided in Table 1. A backward elimination procedure was used, and only covariates with a *p* > 0.15 were retained in the model. In the reporting of results, *p*-values were given for relationships with *p* < 0.05 and estimates of odds ratios together with 95% confidence intervals (95% CI). Where more than one covariate was retained in the model, the *p*-value for the overall model based on the Wald chi-square test statistic was also given.

**TABLE 1a:** Demographic & clinical characteristics of 214 familial and 170 sporadic participants with a DSM diagnosis of schizophrenia or schizoaffective disorder.

Variable	Familial SCZ (*N* = 149)		Sporadic SCZ (*N* = 130)		Familial SAD, bipolar type (*N* = 44)		Sporadic SAD, bipolar type (*N* = 22)		Familial SAD, depressive type (*N* = 21)		Sporadic SAD, depressive type (*N*= 18)	
	Mean	SD	Mean	SD	Mean	SD	Mean	SD	Mean	SD	Mean	SD
Age at recruitment	35.21	13.89	31.16	9.84	38.00	12.83	31.77	10.39	35.48	13.44	29.56	9.24

SCZ, schizophrenia; SAD, schizoaffective disorder; *N*, total number of subjects in a group; *N_c_*, number of subjects in a group for whom the covariate was known; *n*, number of subjects who were positive for a particular covariate; SD, standard deviation.

**TABLE 1b:** Demographic & clinical characteristics of 214 familial and 170 sporadic participants with a DSM diagnosis of schizophrenia or schizoaffective disorder.

Variable	Familial SCZ (*N* = 149)		Sporadic SCZ (*N* = 130)		Familial SAD, bipolar type (*N* = 44)		Sporadic SAD, bipolar type (*N* = 22)		Familial SAD, depressive type (*N* = 21)		Sporadic SAD, depressive type (*N* = 18)	
	%	*n*	%	*n*	%	*n*	%	*n*	%	*n*	%	*n*
Male	59.73	89	70.77	92	54.55	24	59.09	13	61.9	13	61.11	11
Single (Never married)	69.13	103	75.38	98	45.45	20	68.18	15	61.90	13	66.67	12
Employed	82.55	123	82.31	107	68.18	30	63.64	14	47.62	10	72.22	13
Completed Grade 12	44.30	66	64.62	84	68.18	30	86.36	19	66.67	14	66.67	12
Completed tertiary education	12.75	19	12.31	16	25.00	11	18.18	4	19.05	4	27.78	5

SCZ, schizophrenia; SAD, schizoaffective disorder; *N*, total number of subjects in a group; *N_c_*, number of subjects in a group for whom the covariate was known; *n*, number of subjects who were positive for a particular covariate; SD, standard deviation.

**TABLE 1c:** Demographic & clinical characteristics of 214 familial and 170 sporadic participants with a DSM diagnosis of schizophrenia or schizoaffective disorder.

Syndrome features	Familial SCZ (*N* = 149)		Sporadic SCZ (*N* = 130)		Familial SAD, bipolar type (*N* = 44)		Sporadic SAD, bipolar type (*N* = 22)		Familial SAD, depressive type (*N* = 21)		Sporadic SAD, depressive type (*N* = 18)	
	Mean	SD	Mean	SD	Mean	SD	Mean	SD	Mean	SD	Mean	SD
Age of primary diagnosis onset	22.49	7.45	23.29	6.66	25.07	8.06	20.18	3.54	24.62	7.48	21.72	5.38

SCZ, schizophrenia; SAD, schizoaffective disorder; *N*, total number of subjects in a group; *N_c_*, number of subjects in a group for whom the covariate was known; *n*, number of subjects who were positive for a particular covariate; SD, standard deviation.

**TABLE 1d:** Demographic & clinical characteristics of 214 familial and 170 sporadic participants with a DSM diagnosis of schizophrenia or schizoaffective disorder.

Co-morbid diagnosis	Familial SCZ (*N* = 149)		Sporadic SCZ (*N* = 130)		Familial SAD, bipolar type (*N* = 44)		Sporadic SAD, bipolar type (*N* = 22)		Familial SAD, depressive type (*N* = 21)		Sporadic SAD, depressive type (*N* = 18)	
	%	*n*	%	*n*	%	*n*	%	*n*	%	*n*	%	*n*
Intellectual disability	6.04	9	3.85	5	0.00	0	0.00	0	0.00	0	0.00	0
Borderline intellectual disability	8.05	12	3.85	5	4.55	2	0.00	0	0.00	0	0.00	0
Obsessive compulsive disorder	4.70	7	6.15	8	0.00	0	9.09	2	19.05	4	16.67	3
Obsessive compulsive symptoms	4.03	6	6.15	8	2.27	1	4.55	1	9.52	2	0.00	0
Major depressive disorder	10.07	15	9.23	12	15.91	7	9.09	2	19.05	4	27.78	5
Social phobia	2.01	3	0.00	0	0.00	0	4.55	1	4.76	1	11.11	2
Alcohol abuse	12.08	18	13.08	17	25.00	11	9.09	2	19.05	4	33.33	6
Cannabis abuse	34.23	51	35.38	46	27.27	12	36.36	8	66.67	14	44.44	8
> 1 illicit substance used before	10.07	15	12.31	16	11.36	5	9.09	2	4.76	1	27.78	5

SCZ, schizophrenia; SAD, schizoaffective disorder; *N*, total number of subjects in a group; *N_c_*, number of subjects in a group for whom the covariate was known; *n*, number of subjects who were positive for a particular covariate; SD, standard deviation.

**TABLE 1e:** Demographic & clinical characteristics of 214 familial and 170 sporadic participants with a DSM diagnosis of schizophrenia or schizoaffective disorder.

Deviant behavior < age 10	Familial SCZ (*N* = 149)		Sporadic SCZ (*N* = 130)		Familial SAD, bipolar type (*N* = 44)		Sporadic SAD, bipolar type (*N* = 22)		Familial SAD, depressive type (*N* = 21)		Sporadic SAD, depressive type (*N* = 18)	
	%	*n/N_c_*	%	*n/N_c_*	%	*n/N_c_*	%	*n/N_c_*	%	*n*/*N*_c_	%	*n*/*N*_c_
Social difficulties	55.28	68/123	44.25	50/113	50.00	18/36	33.33	7/21	66.67	12/18	64.71	11/17
Extreme aggression	9.40	11/117	6.25	7/112	11.43	4/35	9.52	2/21	0.00	0/16	5.88	1/17
Odd behavior	16.95	20/118	9.91	11/111	5.71	2/35	28.57	6/21	18.75	3/16	11.76	2/17
Extreme fears	24.58	29/118	25.89	29/112	31.43	11/35	19.05	4/21	56.25	9/16	41.18	7/17
Chronic sadness	11.11	13/117	9.09	10/110	20.00	7/35	9.52	2/21	31.25	5/16	23.53	4/17
Learning disability	36.97	44/119	28.57	32/112	20.00	7/35	14.29	3/21	31.25	5/16	35.29	6/17
Impairment in attention	59.83	70/117	49.56	56/113	37.14	13/35	38.10	8/21	68.75	11/16	58.82	10/17
Presence of any early deviant behavior	83.33	105/126	71.93	82/114	75.00	27/36	71.43	15/21	100.00	18/18	82.35	14/17

SCZ, schizophrenia; SAD, schizoaffective disorder; *N*, total number of subjects in a group; *N_c_*, number of subjects in a group for whom the covariate was known; *n*, number of subjects who were positive for a particular covariate; SD, standard deviation.

**TABLE 1f:** Demographic & clinical characteristics of 214 familial and 170 sporadic participants with a DSM diagnosis of schizophrenia or schizoaffective disorder.

Variable	Familial SCZ (*N* = 149)		Sporadic SCZ (*N* = 130)		Familial SAD, bipolar type (*N* = 44)		Sporadic SAD, bipolar type (*N* = 22)		Familial SAD, depressive type (*N* = 21)		Sporadic SAD, depressive type (*N* = 18)	
	Mean	*N_c_*	Mean	*N_c_*	Mean	*N_c_*	Mean	*N_c_*	Mean	*N_c_*	Mean	*N_c_*
Number of early deviant behaviors	2.09	117	1.68	110	1.74	35	1.52	21	2.69	16	2.41	17

SCZ, schizophrenia; SAD, schizoaffective disorder; *N*, total number of subjects in a group; *N_c_*, number of subjects in a group for whom the covariate was known; *n*, number of subjects who were positive for a particular covariate; SD, standard deviation.

**TABLE 1g:** Demographic & clinical characteristics of 214 familial and 170 sporadic participants with a DSM diagnosis of schizophrenia or schizoaffective disorder.

Symptom history	Familial SCZ (*N* = 149)		Sporadic SCZ (*N* = 130)		Familial SAD, bipolar type (*N* = 44)		Sporadic SAD, bipolar type (*N* = 22)		Familial SAD, depressive type (*N* = 21)		Sporadic SAD, depressive type (*N* = 18)	
	%	*n*/*N*_c_	%	*n*/*N*_c_	%	*n*/*N*_c_	%	*n*/*N*_c_	%	*n*/*N*_c_	%	*n*/*N*_c_
Delusions	98.62	143/145	98.46	128/130	100.00	44/44	100.00	22/22	100.00	20/20	100.00	18/18
Hallucinations	94.93	131/138	83.90	99/118	77.50	31/40	95.45	21/22	84.21	16/19	88.24	15/17
Auditory hallucinations	90.15	119/132	80.00	92/115	71.79	28/39	77.27	17/22	78.95	15/19	76.47	13/17
Visual hallucinations	35.43	45/127	36.36	40/110	30.56	11/36	50.00	11/22	31.58	6/19	17.65	3/17
Sensory hallucinations	30.16	38/126	17.27	19/110	20.59	7/34	23.81	5/21	27.78	5/18	5.88	1/17
Disorganized behavior	78.99	94/119	84.11	90/107	84.38	27/32	90.48	19/21	76.47	13/17	76.92	10/13
Disordered thought	73.68	84/114	74.31	81/109	77.14	27/35	85.00	17/20	94.12	16/17	64.29	9/14
Alogia	41.44	46/111	56.73	59/104	45.45	15/33	60.00	12/20	94.74	18/19	71.43	10/14

SCZ, schizophrenia; SAD, schizoaffective disorder; *N*, total number of subjects in a group; *N_c_*, number of subjects in a group for whom the covariate was known; *n*, number of subjects who were positive for a particular covariate; SD, standard deviation.

**TABLE 1h:** Demographic & clinical characteristics of 214 familial and 170 sporadic participants with a DSM diagnosis of schizophrenia or schizoaffective disorder.

Other	Familial SCZ (*N* = 149)		Sporadic SCZ (*N* = 130)		Familial SAD, bipolar type (*N* = 44)		Sporadic SAD, bipolar type (*N* = 22)		Familial SAD, depressive type (*N* = 21)		Sporadic SAD, depressive type (*N* = 18)	
	%	*n/N_c_*	%	*n/N_c_*	%	*n/N_c_*	%	*n/N_c_*	%	*n/N_c_*	%	*n*/*N*_c_
Suicidal ideation	8.78	13/148	9.30	12/129	18.18	8/44	9.09	2/22	28.57	6/21	22.22	4/18
Suicide attempts	22.97	34/148	16.28	21/129	25.00	11/44	36.36	8/22	28.57	6/21	33.33	6/18
Birth and perinatal insults	39.05	41/105	41.44	46/111	27.03	10/37	33.33	7/21	38.89	7/18	30.77	4/13
Winter/spring birth	24.16	36/149	23.85	31/130	15.91	7/44	27.27	6/22	33.33	7/21	11.11	2/18

SCZ, schizophrenia; SAD, schizoaffective disorder; *N*, total number of subjects in a group; *N_c_*, number of subjects in a group for whom the covariate was known; *n*, number of subjects who were positive for a particular covariate; SD, standard deviation.

Two main logit models were considered to account for the large differences in group sizes of primary diagnosis. In the first model (results given in Table 1-A1), the primary diagnosis entered into the model was schizophrenia and schizoaffective disorder (bipolar and depressive types combined) and in the second model (results in Table 2-A1) only schizoaffective disorder was considered and entered as bipolar or depressive. As there are two models for each binary outcome, a Bonferroni correction should be made by comparing the *p*-values given in the table with 0.025 instead of 0.05. General linear models (GLM) were fitted to determine whether any of the covariates (same as for the logit model) are significantly related to the mean age at onset. The normality assumption was validated and Least Squares Mean (LSMeans) was calculated to adjust for the effect of other covariates in the model and differences in group sizes. The results are given in Table 3-A1, and because there are three models, a Bonferroni correction should be made by comparing *p*-values given with 0.05/3 = 0.0167. Tables 1-A1, 2-A1 and 3-A1 are included in Appendix 1.

### Ethical considerations

Consent for the use of information in the future studies was obtained during the initial recruitment. The study protocol was submitted to Master of Medicine (MMed) and Research Ethics Committees of the Faculty of Health Sciences of the University of Pretoria. Written approval was obtained from both committees (Approval number 291/2016).

### Results

Of the 384 participants, 279 (73%) had a diagnosis of schizophrenia, 66 (17%) had a diagnosis of schizoaffective disorder, bipolar type, and 39 (10%) had a diagnosis of schizoaffective disorder, depressive type; 242 (63%) were male and 142 (37%) were female participants. The mean age at onset of the primary diagnosis was 23 years (standard deviation [SD] = 7); 238 (62%) participants had at least one comorbid diagnosis; 45 participants (12%) had suicidal ideation and 86 (22%) had suicide attempts; 115 participants (38%) had birth or perinatal complications; 89 (23%) were born in the winter or spring months (July to September in the southern hemisphere); and 261 participants (79%) had one or more of the seven early childhood deviant behaviours that were assessed. Specifically, 166 participants (51%) had early social difficulties: 25 (8%) had extreme aggression, 44 (14%) had odd behaviour, 89 (28%) had extreme fears, 41 (13%) had chronic sadness, 97 (30%) had a learning disability and 168 (53%) had impairment in attention.

Initial comparisons across the sample, controlling for familiality and diagnosis, mainly identified some gender differences. In the combined schizophrenia and schizoaffective group, when controlling for diagnosis, the odds of being a male subject and single was four times higher compared to female participants (95% confidence interval [CI] = 2.5, 6.3; *p* < 0.0001), an association driven mostly by the schizoaffective disorder bipolar type group, where the corresponding odds ratio was 4.6 (95% CI = 2.0, 10.8; *p* = 0.0004). Another difference in the combined schizophrenia and schizoaffective group was that male participants had higher odds for a comorbid diagnosis than female participants (2.97 [95% CI = 1.9, 4.6; *p* < 0.0001]) and were also more likely to have abused cannabis than female participants (5.8 [95% CI = 3.3, 10.2; *p* < 0.0001]). This association held true when prior abuse of any substance (not just cannabis) was considered (5.2 [95% CI = 3.1, 8.6; *p* < 0.0001]).

We then focused on familiality in the logit models and controlled for sex and diagnosis.

Table 1 shows the descriptive statistics of the three diagnostic groups subdivided into familial and sporadic cases. The table contains information on basic socio-demographic variables, age of primary diagnosis onset, comorbid diagnoses, early childhood deviant behaviours, clinical symptoms history, suicidal ideation or attempts, birth and perinatal insults, as well as season (month) of birth.

From a general linear model, the mean age at onset for sporadic schizophrenia versus familial schizophrenia when controlling for sex did not differ significantly (23.7 vs. 22.7; *p* = 0.23). The adjusted or LSMeans that was calculated also accounted for differences in group sizes. The same was true for schizoaffective disorder, depressive type (22.1 vs. 25.0; *p* = 0.17). However, there was a statistically significant difference in schizoaffective disorder bipolar type, where disease onset was at 20.6 years of age in sporadic cases versus 25.3 years of age in familial cases (*p* = 0.0077, effect size *η*^2^ = 0.23). The difference was also gender-dependent (20.4 for male participants and 25.5 for female participants, *p* = 0.0022).

No statistically significant differences were identified regarding familiality and being single.

There were no differences between sporadic and familial cases with regard to the presence of a comorbid diagnosis in any of the diagnostic groups.

There were no statistically significant differences when comparing birth and perinatal insults based on familiality.

In the combined schizophrenia and schizoaffective disorder group, the odds for the presence of any early deviant behaviour was 1.9 times higher in the familial compared to the sporadic group (95% CI = 1.1, 3.3, *p* = 0.0167) when controlling for sex (Wald chi-square statistic for overall model *p* = 0.02).

Suicidal ideation and suicide attempts were significantly higher (2.3 times) in the schizoaffective disorder group compared to the schizophrenia group (95% CI = 1.5, 3.7, *p* = 0.0003), but there was no significant difference based on familiality.

Frequencies for the month of birth were higher in March, April, May, July, September and October in the familial group, and higher in June in the sporadic group. Further analysis did not yield significant results regarding the season of birth.

## Discussion

We attempted to delineate a more homogeneous phenotype for genetic research via a comparison of various socio-demographic and clinical variables in an adult population with a lifetime diagnosis of schizophrenia or schizoaffective disorder. The main comparative variable was familiality, as this could have significant discriminatory value in genetic studies. Our subjects were drawn from the Afrikaner population in South Africa, where determination of familiality could be carried out relatively accurately given the close-knit family structure, restricted population movement and wide availability of family medical records in long-term mental health facilities.

Our findings demonstrated that the combined schizophrenia and schizoaffective disorder group did not differ significantly on most of the covariates that we compared between the sporadic and familial groups. These included marital status, clinical symptomatology, comorbidities, birth and perinatal insults, suicidality and season of birth. The only variable that differed was the early childhood deviant behaviour, previously linked to the later development of schizophrenia,^13^ which we found to be more common in the familial group. This could make sense from a genetic point of view where familial predisposition could lead to early manifestations of an illness. Early deviant behaviour was not, however, associated with lower age at onset.

When considering the schizoaffective group, we found that the age at onset in the bipolar type of the schizoaffective diagnosis was significantly lower in the sporadic group. Age at onset has been associated with increased genetic loading.^15^ It is also one of the most frequently studied outcome predictors in schizophrenia. A recent meta-analysis found a statistically significant association between lower age at onset and higher rates of hospitalisation, relapses and negative symptoms. It was also associated with worse social or occupational functioning and worse global outcome.^16^ Our study did not specifically look at the number of hospitalisations, relapses, social or occupation functioning or global outcomes in any of the groups.

## Limitations

The study has some limitations. The subjects in the study were all Caucasian of European descent (Afrikaner), and the findings may therefore not be applicable to other races. Although appropriate statistical corrections were made, the diagnostic, gender and familial–sporadic groups were not equally represented. The duration of illness may have also influenced severity and thus presentation at recruitment. The database of information was not complete for all the covariates that were assessed.

The size of the schizoaffective disorder subset also limited the analyses that could be performed. Accurate recording of family history was challenging, and although it was much more accurate in the Afrikaner, and perhaps other founder populations, than outbred populations, there might still be some errors.

## Conclusion

The sporadic bipolar type of the schizoaffective diagnosis seems to form a more homogeneous group with lower age at onset. The age at onset has prognostic value with a possible genetic origin. An earlier age at onset has also been linked to a higher genetic load in Alzheimer’s disease, systemic lupus erythematosus and cancer.^1^

In addition to this study, genetic linkage analysis also appears to support a relatively specific genetic component in schizoaffective disorder.^3^ Specifically, we have previously shown that a genetic linkage to the *13q locus* was 4.16 times more common in schizoaffective disorder compared with linkage to the *1p locus*.^17^

Therefore, the sporadic bipolar type of the schizoaffective diagnosis may be helpful in elucidating the genetic complexity of schizophrenia and should be studied as a separate diagnostic entity in genetic studies.

Unfortunately, there is low diagnostic reliability for the schizoaffective disorder diagnosis^18^ and, in particular, the distinction between the bipolar and the depressive types. Equally challenging is the accurate assessment of the sporadic versus the familial nature of psychiatric illness. Founder populations offer certain advantages on that front.

Given these results, it appears that the efforts required for both correct diagnostic assessments and family history assessments are warranted, as the bipolar type of the schizoaffective diagnosis in its sporadic form is a clinical entity with potential utility in genetic research.

## Appendix 1

**TABLE 1-A1:** Logit models where predictors entered are differential family history, sex, diagnosis (schizophrenia or schizoaffective disorder).

Binary outcome	Analysis of maximum likelihood estimates						Odds ratio estimates				Global null hypothesis		
Outcome = Yes is modelled	Parameter	DF	Est	SE	Wald	*p*	Effect	OR	95% Conf Lim		Wald	DF	*p*
Comorbid diagnosis	Intercept	1	0.3748	0.1103	11.5531	0.0007	-	-	-		24.3535	1	< 0.0001
	Sex	1	0.5442	0.1103	24.3535	< 0.0001	M vs. F	2.970	1.927	4.575	-	-	-
Cannabis use	Intercept	1	−1.0476	0.1416	54.7062	< 0.0001	-	-	-	-	38.7720	1	< 0.0001
	Sex	1	0.8819	0.1416	38.7720	< 0.0001	M vs. F	5.835	3.349	10.166	-	-	-
Illicit substance abuse	Intercept	1	−0.7715	0.1291	35.7053	< 0.0001	-	-	-	-	40.4437	1	< 0.0001
	Sex	1	0.8211	0.1291	40.4437	< 0.0001	M vs. F	5.166	3.114	8.570	-	-	-
Single	Intercept	1	0.5161	0.1260	16.7703	< 0.0001	-	-	-	-	41.0772	2	< 0.0001
	Sex	1	0.6951	0.1166	35.5317	< 0.0001	M vs. F	4.015	2.542	6.342	-	-	-
	Diagnosis	1	−0.3148	0.1258	6.2595	0.0124	SCZ vs. SAD	1.876	1.145	3.077	-	-	-
Perinatal insults	Intercept	1	−0.5772	0.1260	20.9828	< 0.0001	-	-	-	-	4.0357	1	0.0445
	Sex	1	0.2531	0.1260	4.0357	0.0445	M vs. F	1.659	1.012	2.719	-	-	-
Hallucinations	Model not significant												
Early deviant behavior	Intercept	1	1.2534	0.1378	82.6978	< 0.0001					7.6209	2	0.0221
	DFH	1	0.3274	0.1368	5.7262	0.0167	Fam vs. Spor	1.925	1.126	3.290	-	-	-
	Sex	1	0.2249	0.1383	2.6439	0.1039	-	-	-	-	-	-	-
Suicidality	Intercept	1	−0.4791	0.118	16.4865	< 0.0001	-	-	-	-	12.7885	1	0.0003
	Diagnosis	1	0.4220	0.118	12.7885	0.0003	SAD vs. SCZ	2.326	1.464	3.694	-	-	-

DFH, differential family history; DF, degrees of freedom; Est, parameter estimate; SE, standard error; Wald, Wald chi-square statistic; OR, odds ratio; M, male; F, female; SCZ, schizophrenia; SAD, schizoaffective disorder.

**TABLE 2-A1:** Logit models where predictors entered are DFH, sex, schizoaffective disorder (bipolar or depressive).

Binary outcome	Analysis of maximum likelihood estimates						Odds ratio estimates				Global null hypothesis		
Outcome = Yes is modelled	Parameter	DF	Est	SE	Wald	*p*	Effect	OR	95% Conf Lim		Wald	DF	*p*
Comorbid diagnosis	Intercept	1	0.4039	0.2192	3.3957	0.0654	-	-	-	-	7.6387	2	0.0219
	Sex	1	0.4821	0.2075	5.3993	0.0201	M vs. F	2.623	1.163	5.915	-	-	-
	SAD (b or d)	1	−0.3361	0.2185	2.3669	0.1239	-	-	-	-	-	-	-
Cannabis use	Intercept	1	−1.0088	0.2585	15.2269	< 0.0001	-	-	-	-	11.2095	2	0.0037
	DFH	1	0.3732	0.2288	2.6597	0.1029	M vs. F	4.786	1.739	13.172	-	-	-
	Sex	1	0.7829	0.2583	9.1884	0.0024	-	-	-	-	-	-	-
Illicit substance abuse	Intercept 1	1	−0.4183	0.2227	3.5271	0.0604	-	-	-	-	10.3389	2	0.0057
	DFH	1	−0.3987	0.2147	3.4488	0.0633	M vs. F	3.371	1.421	7.997	-	-	-
	Sex	1	0.6076	0.2203	7.6052	0.0058	-	-	-	-	-	-	-
Single	Intercept	1	0.2941	0.2243	1.7185	0.1899	-	-	-	-	14.3893	2	0.0008
	DFH	1	−0.3754	0.2249	2.7856	0.0951	M vs. F	4.641	1.990	10.823	-	-	-
	Sex	1	0.7674	0.2160	12.619	0.0004	M vs. F	3.789	1.349	10.643	-	-	-
Perinatal insults	Intercept	1	−0.9762	0.2635	13.7255	0.0002	-	-	-	-	6.3893	1	0.0115
	Sex	1	0.6660	0.2635	6.3893	0.0115	-	-	-	-	-	-	-
Hallucinations	Intercept	1	−1.5785	0.2977	28.1059	< 0.0001	Fam vs. Spo	3.446	1.073	11.072	4.3178	1	0.0377
	DFH	1	0.6187	0.2977	4.3178	0.0377	-	-	-	-	-	-	-
Early deviant behavior	Intercept	1	1.6983	0.3373	25.3551	< 0.0001	-	-	-	-	3.9304	1	0.0474
	SAD (b or d)	1	−0.6686	0.3373	3.9304	0.0474	D vs. B	3.802	1.015	14.286	-	-	-

DFH, differential family history ; DF, degrees of freedom; Est, parameter estimate; SE, standard error; Wald, Wald chi-square statistic; OR, odds ratio; SAD (b or d), schizoaffective disorder (bipolar or depressive); M, male; F, female.

Note: Suicidality, model not significant.

**TABLE 3-A1:** General linear models for different diagnosis; age at onset as outcome variable and predictors entered are differential family history and sex.

Diagnosis	Analysis of maximum likelihood estimates					LSMeans					
	Parameter	Est	SE	*t*-value	*p*	DFH	LSMeans	*p*	Sex	LSMeans	*P*
Schizophrenia	Intercept	24.7081	0.8818	28.02	< 0.0001	Familial	22.7	0.2302	Male	22.7	0.0252
	DFH	0.4821	0.2075	−1.20	0.2302	Sporadic	23.7	-	Female	24.2	-
	Sex	−0.3361	0.2185	−2.25	0.0252	-	-	-	-	-	-
Schizoaffective disorder bipolar type	Intercept	23.2042	1.6738	13.86	< 0.0001	Familial	25.3	-	Male	20.4	0.0022
	DFH	4.6539	1.6904	2.75	0.0077	Sporadic	20.6	0.0077	Female	25.5	-
	Sex	−5.1148	1.6056	−3.19	0.0022	-	-	-	-	-	-
Schizoaffective disorder depressive type	Intercept	23.5755	2.0172	11.69	< 0.0001	Familial	25.0	0.1710	Male	22.0	0.1656
	DFH	2.9209	2.0911	1.40	0.1710	Sporadic	22.1	-	Female	25.0	-
	Sex	−3.0327	2.1428	−1.42	0.1656	-	-	-	-	-	-

DFH, differential family history; DF, degrees of freedom; Est, parameter estimate; SE, standard error; LSMeans, least squares means.
